# Supplementary material for: Longitudinal high-throughput TCR repertoire profiling reveals the dynamics of T-cell memory formation after mild COVID-19 infection
Source: eLife. 2021 Jan 5;10:e63502. doi: 10.7554/eLife.63502 (PMC7806265; doi:10.7554/eLife.63502)
Supplement: Transparent reporting form [file elife-63502-transrepform.docx]

***eLife’s* transparent reporting form**

We encourage authors to provide detailed information *within their submission* to facilitate the interpretation and replication of experiments. Authors can upload supporting documentation to indicate the use of appropriate reporting guidelines for health-related research (see [EQUATOR Network](http://www.equator-network.org/%20)), life science research (see the [BioSharing Information Resource](https://biosharing.org/)), or the [ARRIVE guidelines](http://www.plosbiology.org/article/info:doi/10.1371/journal.pbio.1000412) for reporting work involving animal research. Where applicable, authors should refer to any relevant reporting standards documents in this form.

If you have any questions, please consult our Journal Policies and/or contact us: [editorial@elifesciences.org](mailto:editorial@elifesciences.org).

**Sample-size estimation**

- You should state whether an appropriate sample size was computed when the study was being designed
- You should state the statistical method of sample size computation and any required assumptions
- If no explicit power analysis was used, you should describe how you decided what sample (replicate) size (number) to use

Please outline where this information can be found within the submission (e.g., sections or figure legends), or explain why this information doesn’t apply to your submission:

Current research is focused on deep sequencing of individual TCR alpha and beta repertoires after SARS-CoV-2 infection, rather than measuring a single statistic for large groups of donors. The timepoints for sampling blood were selected according to T cell response in mild COVID-19 dynamics shown in [Thevarajan et al. 2020] as well as in human acute viral infection models [Miller et al. 2008]: day 15 after exposure to the virus should be close to the peak of T cell response, and day 85 timepoint is late enough to capture virus-specific clonotypes contraction after acute infection [Pogorelyy et al. 2018]. The list of timepoints and samples used could be found in SI Table 1.

**Replicates**

- You should report how often each experiment was performed
- You should include a definition of biological versus technical replication
- The data obtained should be provided and sufficient information should be provided to indicate the number of independent biological and/or technical replicates
- If you encountered any outliers, you should describe how these were handled
- Criteria for exclusion/inclusion of data should be clearly stated
- High-throughput sequence data should be uploaded before submission, with a private link for reviewers provided (these are available from both GEO and ArrayExpress)

Please outline where this information can be found within the submission (e.g., sections or figure legends), or explain why this information doesn’t apply to your submission:

Two biological replicates (two independent portions of blood) were used for bulk post-infection PBMC samples (first paragraph of Results, Fig. 1 caption and SI Table 1). All other samples were collected and sequenced without biological replicates (SI Table 1). Raw high-throughput sequencing data was deposited to SRA, accession number PRJNA633317.

**Statistical reporting**

- Statistical analysis methods should be described and justified
- Raw data should be presented in figures whenever informative to do so (typically when N per group is less than 10)
- For each experiment, you should identify the statistical tests used, exact values of N, definitions of center, methods of multiple test correction, and dispersion and precision measures (e.g., mean, median, SD, SEM, confidence intervals; and, for the major substantive results, a measure of effect size (e.g., Pearson's r, Cohen's d)
- Report exact p-values wherever possible alongside the summary statistics and 95% confidence intervals. These should be reported for all key questions and not only when the p-value is less than 0.05.

Please outline where this information can be found within the submission (e.g., sections or figure legends), or explain why this information doesn’t apply to your submission:

1) Fig 1 bc, N, mean and SE are indicated in Fig. 1 caption (p.3)
2) Fig. 1de, Fig. 2-4, Fig. S3cd Fig. S6 and Fig. S7, N of expanded/contracted clonotypes is shown in the main text (p. 2, right column), statistical test to identify contracted/expanded clonotypes, significance threshold and multiple testing correction technique are described in methods (p.8, right column)
3) Total number of cells, unique clonotypes and UMIs could be found in SI Table 1.
4) Fig. S3ab, N, mean and SE are indicated in figure 1 caption (p.9)
5) Fig. S4, number of significantly expanded/contracted clones as detected by edgeR and NoisET are indicated in Venn diagrams on the Fig. S4b,c,e,f significance threshold for NoisET method could be found in methods (p.8, right column)

(For large datasets, or papers with a very large number of statistical tests, you may upload a single table file with tests, Ns, etc., with reference to sections in the manuscript.)

**Group allocation**

- Indicate how samples were allocated into experimental groups (in the case of clinical studies, please specify allocation to treatment method); if randomization was used, please also state if restricted randomization was applied
- Indicate if masking was used during group allocation, data collection and/or data analysis

Please outline where this information can be found within the submission (e.g., sections or figure legends), or explain why this information doesn’t apply to your submission:

This is a case study, so samples were not allocated into experimental groups.

**Additional data files (“source data”)**

- We encourage you to upload relevant additional data files, such as numerical data that are represented as a graph in a figure, or as a summary table
- Where provided, these should be in the most useful format, and they can be uploaded as “Source data” files linked to a main figure or table
- Include model definition files including the full list of parameters used
- Include code used for data analysis (e.g., R, MatLab)
- Avoid stating that data files are “available upon request”

Please indicate the figures or tables for which source data files have been provided:

SI Table 3 contains list of contracted TCRbeta clonotypes with their concentration on each timepoint, presence in HLA-A02-YLQPRTFLL tetramer+ repertoire, mapped cognate peptides from MIRA assay and predicted TCRalpha chain.

SI Table 4 contains list of contracted TCRalpha clonotypes with their concentration on each timepoint, presence in HLA-A02-YLQPRTFLL tetramer+ repertoire.

SI Table 5 contains list of expanded TCRbeta clonotypes with their concentration on each timepoint, presence in HLA-A02-YLQPRTFLL tetramer+ repertoire, mapped cognate peptides from MIRA assay and predicted TCRalpha chain.

SI Table 6 contains list of expanded TCRalpha clonotypes with their concentration on each timepoint, presence in HLA-A02-YLQPRTFLL tetramer+ repertoire.

All processed repertoire data and lists of significantly expanded/contracted TCR clonotypes are available on the GitHub (<https://github.com/pogorely/Minervina_COVID)>, this link could be found in “Data availability” Methods section. These processed datasets are sufficient to reproduce main text Fig. 1-4.
